# A New Easy-to-Perform Flow Cytometry Assay for Determining Bacterial- and Viral-Infection-Induced Polymorphonuclear Neutrophil and Monocyte Membrane Marker Modulation in Febrile Patients

**DOI:** 10.3390/ijms252111632

**Published:** 2024-10-29

**Authors:** Marilena La Sorda, Desy De Lorenzis, Alessandra Battaglia, Barbara Fiori, Rosalia Graffeo, Rosaria Santangelo, Tiziana D’Inzeo, Gennaro De Pascale, Giovanni Schinzari, Romina Rose Pedone, Ernesto Rossi, Maurizio Sanguinetti, Michela Sali, Andrea Fattorossi

**Affiliations:** 1 Department of Laboratory Sciences and Infectious Diseases, A. Gemelli University Hospital Foundation IRCCS, 00168 Rome, Italy; marilena.lasorda@policlinicogemelli.it (M.L.S.); desy.delorenzis@gmail.com (D.D.L.); barbara.fiori@policlinicogemelli.it (B.F.); rosalia.graffeo@policlinicogemelli.it (R.G.); rosaria.santangelo@policlinicogemelli.it (R.S.); tiziana.dinzeo@policlinicogemelli.it (T.D.); maurizio.sanguinetti@policlinicogemelli.it (M.S.); michela.sali@policlinicogemelli.it (M.S.); 2 Department of Life Science and Public Health, Catholic University of the Sacred Heart, 00168 Rome, Italy; alessandra.battaglia@gmail.com; 3 Emergency Department, A. Gemelli University Hospital Foundation IRCCS, 00168 Rome, Italy; gennarodepascale@policlinicogemelli.it; 4 Medical Oncology, A. Gemelli University Hospital Foundation IRCCS, 00168 Rome, Italy; giovanni.schinzari@policlinicogemelli.it (G.S.); rominarose.pedone@guest.policlinicogemelli.it (R.R.P.); ernesto.rossi@policlinicogemelli.it (E.R.); 5 Department of Basic Biotechnological Sciences, Intensive and Perioperative Clinics, Catholic University of the Sacred Heart, 00168 Rome, Italy; 6 Laboratory of Oncology and Flow Cytometry, A. Gemelli University Hospital Foundation IRCCS, 00168 Rome, Italy

**Keywords:** flow cytometry, bacterial and viral infection, polymorphonuclear neutrophils, monocytes, immune dysfunction, interleukin-1 receptor 2, HLA-DR, CD64, CD169

## Abstract

We developed a flow cytometry (FC) assay enabling the rapid and accurate identification of bacterial and viral infections using whole blood samples. The streamlined flow cytometry assay is designed to be user-friendly, making it accessible even for operators with limited experience in FC techniques. The key components of the assay focus on the expression levels of specific surface markers—CD64 on polymorphonuclear neutrophils (PMN) as a marker for bacterial infection, and CD169 on monocytes (MO) for viral infection. The strong performance indicated by an area under the receiver operating characteristic (ROC) curve of 0.94 for both PMN CD64 positive predictive value (PPV) 97.96% and negative predictive value (NPV) 76.67%, and MO CD169 PPV 82.6% and NPV 86.9%, highlight the assay’s robustness in differentiating between bacterial and viral infections accurately. The FC assay includes the assessment of immune system status through HLA-DR and IL-1R2 modulation in MO, providing a useful insight into the patients’ immune response. The significant increase in the frequency of MO exhibiting reduced HLA-DR expression and elevated IL-1R2 levels in infected patients (compared to healthy controls) underscores the potential of these markers as indicators of infection severity. Although the overall correlation between HLA-DR and IL-1R2 expression levels was not significant across all patients, there was a trend in patients with more severe disease suggesting that these markers may have the potential to assist in stratifying patient risk. The present FC assay has the potential to become routine in the clinical microbiology laboratory community and to be helpful in guiding clinical decision making.

## 1. Introduction

Recognizing bacterial and viral etiologies in febrile patients is often a diagnostic challenge [1]. Medical history, physical findings, and ancillary medical tests, such as the assessment of the standard biomarkers of inflammation, including leukocyte count, absolute neutrophil count, and acute phase reactant levels (C-reactive protein, procalcitonin), have a reasonable negative predictive value for infection, but their positive predictive value is limited and does not reliably distinguish between the two different causative agents [2]. This clinical uncertainty may alter the trajectory of patient care, including antibiotic misuse, both over- and under-use, with consequences for individual and global health, including the emergence of antibiotic resistance [3,4]. To facilitate accurate clinical decision making, various microbiological tests are regularly requested. Blood or other bodily fluid cultures are the methods commonly used in clinical settings to identify microbial pathogens, but their diagnostic utility is often reduced by the initiation of empiric antimicrobial treatment, sampling quality, contamination, and, most significantly, long lead times of approximately 24 to 48 h. This length of time before deciding what initial treatment to pursue is usually associated with a worst prognosis [5]. In addition, sensitivity may be reduced if only low sample volumes are available, such as in pediatric patients, and repeated blood samplings are required [6,7]. Perhaps most importantly, these tests are inherently limited by the need to sample the infection focus, which is particularly challenging in lower respiratory infections and fever without source. Therefore, there is a pressing need for reliable and rapid tools to aid clinicians in detecting the presence of a bacterial or viral infection in febrile patients so that immediate and appropriate treatment can be started.

Innate immunity is the first line of defense against pathogen invasion and involves complex and dynamic interplays between pathogens and host immune defense mechanisms. Myeloid cells, such as polymorphonuclear neutrophils (PMN) and monocytes (MO), are the first mechanisms of innate immunity that oppose the spread of pathogenic organisms [8]. Both PMN and MO use pattern recognition receptors to immediately identify the pathogen-associated or damage-associated molecular patterns and produce a robust inflammatory response that leads to activation of several signaling cascades including the production of antibacterial or antiviral mediators and the modulation of various functional molecules on the cell surface [8].

The most intensively investigated surface molecule in PMN as a biomarker of an infection of bacterial origin is Fc-gamma receptor 1—commonly referred to as CD64—a type of integral membrane glycoprotein that binds monomeric IgG-type antibodies with high affinity [9,10]. CD64 molecules are barely present on the membrane of resting PMN, being stored intracellularly, but can be sharply mobilized upon bacterial activation. The surface density of CD64 in PMN is estimated to be approximately < 1000 sites per cell, which is at the lowest level of detection by most flow cytometers. CD64 density increases within 10–120 min after exposure to lipopolysaccharide in vitro and may remain stable for over 48 h [11]. There is also evidence that cytokines such as G-CSF may act on myeloid precursor cells and induce CD64 expression in PMN released into circulation [12,13]. Taken together, these studies imply that CD64 expression on the PMN membrane seen in patients with bacterial infection is a reliable indicator of direct pathogen contact, cytokine exposure, or both.

Numerous preclinical studies have demonstrated the protective role of sialoadhesin or siglec-1 (CD169) expressed on the MO membrane in viral infections. For example, during Friend virus complex infection, CD169 expressing MO reduced viral dissemination in the spleen and induced an effective cytotoxic T lymphocyte response [14]. Similar findings have been described in experimental vesicular stomatitis virus infections [15]. HIV infection has been shown to increase CD169 expression in MO in a non-human primate model [16] and in HIV patients [17]. ZIKV-infected patients also show increased CD169 expression in MO [18].

More recently, CD169 expression in MO has received great attention in the context of SARS-CoV-2 infection and has been shown to be a sensitive biomarker for the early diagnosis and follow up of COVID-19 patients [19,20,21,22,23]. Mechanistic studies have shown that CD169 expression is upregulated by IFN-α [24,25], a cytokine typically released into circulation upon viral infection. In this regard, CD169 modulation has even been proposed as an indirect measure of serum IFN-α level [26]. In addition, MO play various roles during the response to infection that are reflected in the modulation of functional cell surface markers such as HLA-DR and interleukin-1 receptor type 2 (IL-1R2, also known as CD121b). A progressive decrease in HLA-DR expression level in MO is consensually recognized as a reliable marker of infection-evoked immunosuppression and it has been linked to poor prognosis in critically ill patients that experience an initial strong pro-inflammatory phase that then transitions to an anti-inflammatory phase until immunoparalysis [27,28]. IL-1R2 is a high-affinity decoy receptor that participates in the regulation of the inflammatory response by competing with IL-1R1 to bind IL-1 without inducing signaling in that it acts as the negative regulator of the proinflammatory IL-1 signaling pathway [29]. As a conserved immune evasion strategy employed by evolutionary pathogens to limit the effects of cytokines, chemokines, and growth factors [30], it is not surprising that a multiple whole blood gene expression study showed that MO characterized by a high level of IL-1R2 membrane expression appears in the circulation as a consequence of severe bacterial infection [31].

The various studies listed above successfully used flow cytometry (FC) assays to detect the modulation of PMN and MO surface markers to diagnose bacterial and viral infections and infection-induced immune paralysis using fluorochrome-conjugated monoclonal antibodies in whole blood samples [19,20,21,22,23,24,25,26,28,32,33,34,35,36,37]. However, while the modulation of CD64 in PMN is relatively easy to assess, being commonly measured by using lymphocytes, which do not express CD64, as an internal biological control, the extent of the modulation of CD169, HLA-DR, and IL-1R2 in MO is relatively more difficult to measure simply and objectively, as appropriate internal controls have not yet been defined. For example, the measurement of HLA-DR modulation in various bacterial and viral infection contexts to obtain information about the status of the patient’s immune system has led to controversial results that have cast doubt on its diagnostic value [34,35,36]. These discrepancies likely reflect the typical uncertainty in defining the magnitude of changes occurring in cell markers that show a unimodal distribution with no clear separation between positive and negative cells, making data analysis susceptible to inter-user variability. The present FC assay includes the evaluation of IL-1R2 and HLA-DR, both markers that show a unimodal distribution that is difficult to analyze. To overcome this problem, FC assays have been developed that translate flow signal intensity values into antibody binding capacity (ABC) units employing calibrated beads [37]. This method has the obvious advantage of reducing the dependence on operator subjectivity, but it is time-consuming and costly and requires skilled operators, additional reagents, and a longer intervention time, which limit widespread clinical use. Adding to this is the fact that flow cytometers have only just become part of the clinical microbiology laboratory community. Therefore, although FC assays potentially bear all the hallmarks of viable diagnostic tools to complement classical microbiology tests, their use in clinical practice remains limited by these technicalities, and an easy-to-perform and inexpensive FC assay to identify bacterial and viral infections and provide information on the patient’s immune system status through a simple assessment of the modulation of PMN and MO surface marker remains an unmet need.

The goal of this study is to describe an FC assay that builds on pre-existing FC assays exploring the modulation PMN and MO membrane molecules in the context of infectious disease, but minimizes the work up and guesswork inherent in previously published ones and eliminates the need for experienced operators to stain samples and interpret data. In addition, this study explores for the first time the modulation of IL-1R2 in MO in unselected patients with acute infections. These features will likely allow the present FC assay to become a routine procedure in clinical microbiology laboratories and possibly at points of care to guide febrile patient management in a timely manner.

## 2. Results

### 2.1. Gating Strategy and Assessment of PMN and MO Marker Modulation

As a premise of this study, we observed two PMN populations with different forward and side scatter (FSC and SSC, respectively) signal intensities in most blood samples (Supplementary Information, Figure S1A,B). An early study suggested that PMN with a low FSC signal and high SSC signal, such as those we observed here, represented apoptotic PMN [38], a phenomenon that probably reflects a peculiar vulnerability of PMN to unavoidable shear stress during sample processing. The cell membrane of apoptotic cells is altered and may facilitate non-specific antibody binding. Therefore, we first ascertained that the PMN CD64 expression level was not altered in apoptotic PMN. We also verified that apoptotic PMN did not affect the autofluorescence of the total PMN population in the PE channel (Supplementary Information, Figure S1C–E), which is important because PMN are the internal negative control for the PE-conjugated anti-CD169 monoclonal antibody used to measure CD169 expression in MO.

FC data analysis basically relies on the gating strategy to investigate and quantify the populations of interest. Unfortunately, gating is an inherently subjective and error-prone procedure. Therefore, we preferred using rectangular regions, which are easier to place manually around the cell populations of interest, i.e., PMN, MO, Non-B lymphocytes, and B lymphocytes, than polygonal regions to minimize the impact of subjectivity on data analysis and to make the assay as simple as possible for inexperienced users. The gating procedure was further facilitated by visualizing the SSC signal in log mode instead of the more commonly used linear mode. Finally, the present FC assay used the same fluorochrome for CD64 and CD19 to allow operators to draw all regions around the different cell populations of interest on the initial SSC/CD64-CD19 plot.

**Figure 1:**
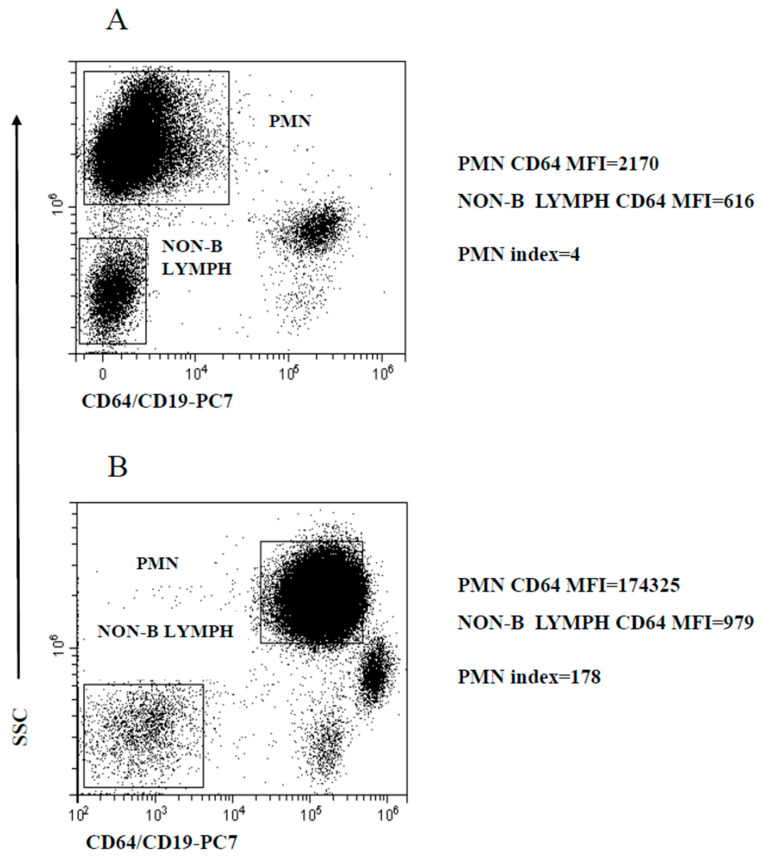
Gating strategy to analyze PMN CD64 expression level modulation. PMN and non-B lymphocyte regions used to calculate the PMN index. (**A**) Representative healthy control; (**B**) microbiologically confirmed bacterial infection patient. PMN and non-B lymphocytes MFI are displayed along with the PMN index.

The activated PMN are homogeneous in terms of CD64 expression. Thus, based on early studies [20,21,22,25,32,33], PMN CD64 modulation was measured as the ratio of mean CD64 expression (calculated as mean fluorescence intensity, MFI) to the MFI of CD64-negative cells, thereafter referred to as PMN index. For this purpose, we preferred to use non-B lymphocytes MFI instead of the more common MFI of the entire population of non-myeloid cells, since preliminary experiments indicated that the autofluorescence of this population was somewhat more consistent across samples. The regions delineated by SSC intensity and CD64/CD19 staining to identify PMN and non-B lymphocytes, the calculated MFI and determined PMN index are illustrated in Figure 1, exemplifying a healthy control (HC) (Figure 1A) and a patient with a microbiologically confirmed bacterial infection (Figure 1B).

CD169, HLA-DR, and IL-1R2 in MO are all characterized by unimodal distribution and homogeneous expression and modulation, similar to that of CD64 in PMN. Although in principle these markers could also be studied by developing the appropriate indices, we thought that this approach would require additional working time. Therefore, we decided to express the modulation of these markers as percent-positive cells to simplify and accelerate data analysis. To this end, we devised a strategy that uses the different cell populations in the whole blood sample as internal negative controls for the visual positioning of the target boundaries. All cell types inherently possess autofluorescence because of the different amounts of naturally occurring fluorochromes, including nicotinamide adenine dinucleotide phosphate (NAD(P)H), flavinins, porphyrin, lipofuscin, and others that are excited by the incident lasers and emit across a wide range of wavelengths [39]. In lysed whole blood samples, PMNs are more autofluorescent than MO and both cell populations are more autofluorescent than lymphocytes. In the present study, the autofluorescence of PMN was ~1.4 higher than the autofluorescence of MO in the PE channel and was therefore deemed acceptable for visual boundary placement for measuring PE-CD169 expression in MO (MO CD169). The autofluorescence of PMNs was only ~1.1 higher than the autofluorescence of MO in the FITC channel, but PMN could not be used as a border for measuring FITC-IL-1R2 expression in MOs (MO IL-1R2) because PMN, as well as B lymphocytes, can express surface IL-1R2 under certain circumstances [40,41]. Therefore, we were forced to use non-B lymphocytes to establish the negative boundary for MO IL-1R2, despite the fact that the autofluorescence of MO in the FITC channel was considerably higher (~1.8) than that of non-B lymphocytes.

**Figure 2:**
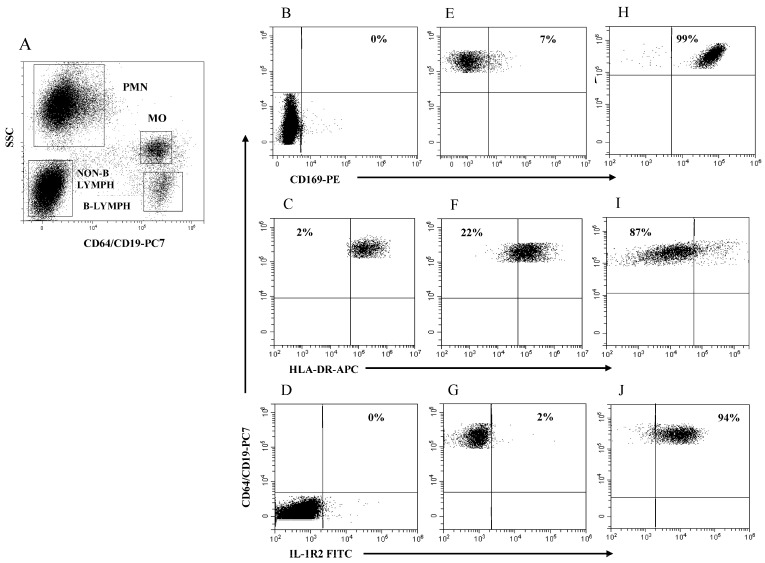
Gating strategy to analyze MO CD169, MO HLA-DR, and MO IL-1R2 modulation. (**A**) Selection of PMN, MO, non-B lymphocytes, and B lymphocytes to establish boundary position for each marker; (**B**) boundary for MO CD169 using PMN; (**C**) boundary for MO HLA-DR using B-lymphocytes; (**D**) boundary for MO IL-1R2 using non-B lymphocytes; (**E**–**G**) representative healthy control; (**H**) patient with SARS-CoV-2 infection; (**I**,**J**) patients with severe bacterial infection. Percentage of cells above or below the boundary is indicated in each plot.

The boundary for HLA-DR expression in MO (MO HLA-DR) was easily set using B lymphocytes as a purely internal HLA-DR-positive reference cell population. Figure 2A illustrates the four rectangular regions delineated by SSC intensity and CD64/CD19 staining to identify the PMN, MO, non-B lymphocytes, and B lymphocytes in a representative HC. Figure 2B–D illustrates the boundaries for measuring the modulation of MO CD169, MO HLA-DR, and MO IL-1R2 using PMN (B), B lymphocytes (C), and non-B lymphocytes (D) as internal references, respectively. Figure 2E–G illustrates MO CD169 (E), MO HLA-DR (F), and MO IL-1R2 (G) expression level as cell percentage in a representative HC. Figure 2H–J exemplifies MO CD169 (H), MO HLA-DR (I), and MO-IL-1R2 (L) modulation in a representative patient with SARS-CoV-2 infection (H) and in critical patients with bacterial (I) and viral infection (L). Note that the modulation MO CD169 and MO IL-1R2 is represented as a percentage of MO above the boundary, while the modulation of MO HLA-DR is represented as a percentage of MO below the boundary. Of note is that, with the obvious exception of the PMN region that had to be extended to accommodate PMN with the increased level of CD64 expression in subjects with bacterial infection, the remaining regions had to be only marginally adapted to encompass the cell cluster of interest throughout the study.

**Figure 3:**
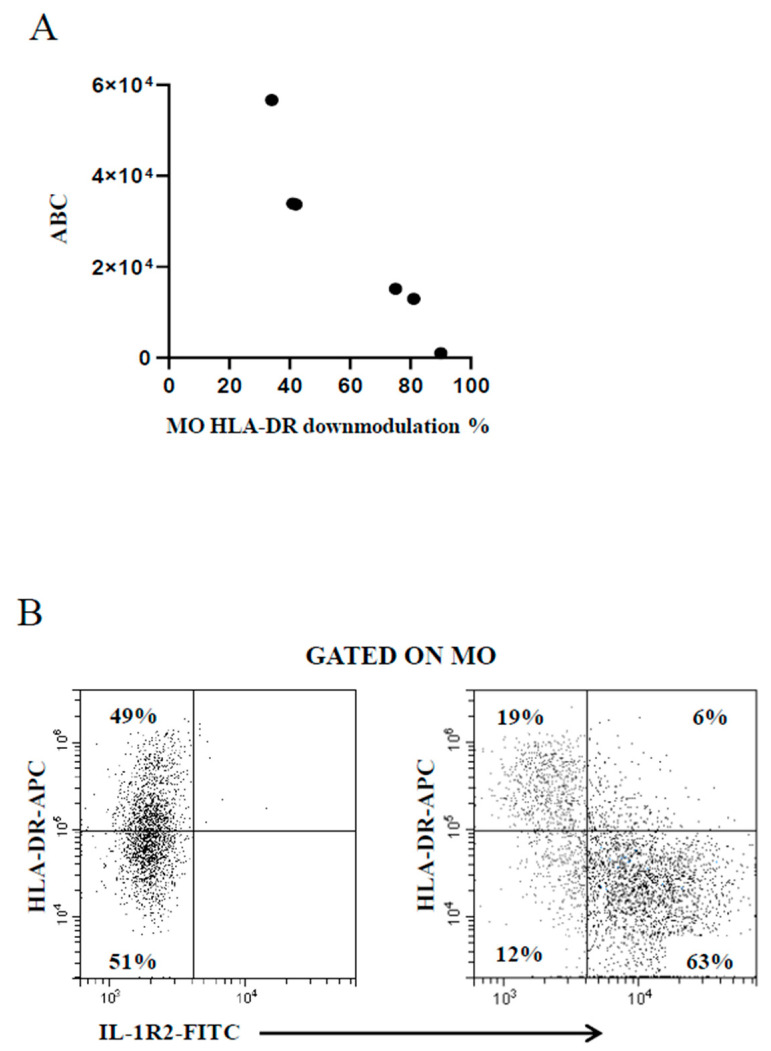
(**A**) Good correspondence between MO HLA-DR downmodulation calculated by the present FC assay (percentage of MO with reduced HLA-DR expression level, *x-axis*) and the Quantibrite™ Anti–HLA-DR/Anti-Monocyte kit (antibody binding capacity, ABC, *y-axis*). (**B**) Concomitant modulation of MO HLA-DR and MO IL-1R2 in a representative healthy control subject (**left plot**) and a critically ill febrile metastatic gastric cancer patient with severe bacterial infection (**right plot**).

The use of the BD Quantibrite™ Anti–HLA-DR/Anti-Monocyte kit is deemed as a trustworthy tool to estimate the number of bound antibodies per cell [37] beyond the inherent variability of the FC assays due to the performance of monoclonals and flow cytometers. Thus, some samples were run in parallel to compare the extent of MO HLA-DR downmodulation measured by the present FC assay with that provided by the kit. Figure 3A shows the good correspondence between data generated by the two assays, confirming the reliability of the present FC assay. A study has shown that MO HLA-DR downmodulation in concomitance with MO IL-1R2 upmodulation is associated with poor prognosis in patients with invasive bacterial infection [31]. The present FC assay was developed to serve as an accessible diagnostic aid in unselected febrile patients regardless of the severity of the pathology in the clinical setting of general hospital. As a result, there were not enough cases of seriously ill patients for statistical analysis to prove or disprove that previous finding [31]. However, a concomitant MO HLA-DR downmodulation and MO IL-1R2 increase was observed in the few patients with more severe infection (Figure 3B).

### 2.2. Modulation of PMN Index and MO Markers

Patients with microbiologically confirmed bacterial and viral infection are thereafter referred to as BaMC and ViMC patients, respectively. Patients in which microbiological confirmation was not obtained due to the physician’s decision are thereafter referred to as without microbiological confirmation (WMC) patients. Non-infected patients with uveal melanoma are thereafter referred to as tumor (T) patients. To illustrate FC assay output in the real-world context, data are reported using box and whisker plots to provide an unobstructed view of the data, and outliers have not been removed.

**Figure 4:**
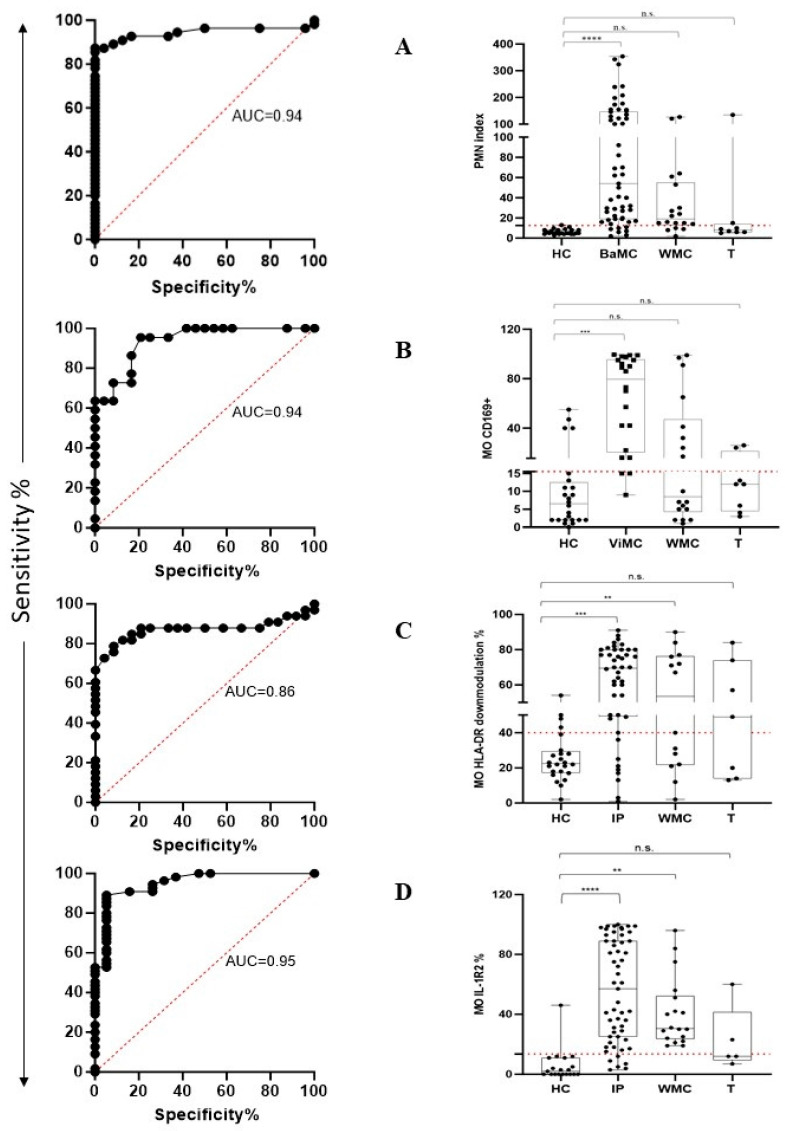
FC assay performance in recognizing patients with bacterial and viral infection according to PMN CD64 and MO CD169 modulation and in stratifying febrile patients into different groups according to MO HLA-DR and MO IL-1R2 expression level. Left plots show the area under the receiver operating characteristic curve (AUC curve). Right plots show data distribution as box plots (median value and interquartile range) and cutoff value (red dotted line). (**A**) PMN index. (**B**) MO CD169. (**C**) MO HLA-DR. (**D**) MO IL-1R2. HC, healthy controls; BaMC and ViMC, febrile patients with microbiologically confirmed bacterial and viral infection, respectively; WMC, febrile patients without available microbiological confirmation; IP, infected patients (cumulated data of BaMC and ViMC patients); T, tumor patients. Statistically significant differences are shown as ** for *p* ≤ 0.02, *** for *p* ≤ 0.001, and **** for *p* ≤ 0.0001; not statistically significant differences are indicated as n.s.

The performance of the PMN index in distinguishing HC from BaMC patients was assessed using receiver operating characteristic (ROC) curve analysis. PMN index values yielded an area under the ROC curve (AUC) of 0.94 (*p* < 0.0001) (Figure 4A, left plot). According to the maximized likelihood ratio, the best cutoff value was 12 with 87.3% sensitivity and 95.8% specificity. Positive predictive value (PPV) and negative predictive value (NPV) were 97.96 and 76.67, respectively. The calculated PMN index in HC, BaMC, WMC, and T patients is reported in Figure 4A, right plot, that also shows the cutoff value. The PMN index was significantly higher in BaMC patients than HC (*p* < 0.0001, one-way ANOVA and Tukey’s test). There was no significant difference between the HC and WMC group. However, MO IL-1R2 upmodulation in the latter group tended to be higher in the few patients that exceeded cutoff, which is suggestive of overlooked bacterial infections. Two T patients had a PMN index above cutoff. The performance of MO CD169 upmodulation in distinguishing HC from ViMC patients was evaluated using ROC curve analysis. The MO CD169 upmodulation yielded an AUC of 0.94 (*p* < 0.0001) (Figure 4B left plot). According to the maximized likelihood ratio, the best cutoff value was 15.5 with 86.4% sensitivity and 83.3% specificity. PPV and NPV were 82.6 and 86.9, respectively. The MO CD169 upmodulation measured in HC, ViMC, WMC, and T patients is reported in Figure 4B right plot, which also shows the cutoff value. The MO CD169 upmodulation was significantly higher in ViMC patients than HC (*p* < 0.001, one-way ANOVA, and Tukey’s test). There was no significant difference between HC and WMC patients. However, a substantial proportion of patients in the WMC group showed MO CD169 upmodulation above cutoff, suggesting an overlooked viral infection. Two T patients had an MO CD169 upmodulation above cutoff. MO HLA-DR downmodulation and MO IL-1R2 upmodulation performance in distinguishing HC from infected patients (for this analysis, BaMC and ViMC data were cumulated and referred to as infected patients, IP) was evaluated using ROC curve analysis. The MO HLA-DR downmodulation yielded an AUC of 0.86 (*p* < 0.0001) (Figure 4C, left plot). According to the maximized likelihood ratio, the best cutoff value was 39.5 with 81.8% sensitivity and 83.3% specificity. The MO HLA-DR downmodulation measured in HC, IP, WMC, and T groups is reported in Figure 4C, right plot, which also shows the cutoff value. The extent of MO HLA-DR downmodulation was significantly higher in IP and WMC groups than HC (*p* < 0.001 and *p* < 0.02, one-way ANOVA and Tukey’s test, respectively). Four out of the seven T patients showed MO HLA-DR downmodulation above the cutoff. The IL-1R2 upmodulation yielded an AUC of 0.95 (*p* < 0.0001) (Figure 4D, left plot). According to the maximized likelihood ratio, the best cutoff value was 13.5 with 89.1% sensitivity and 94.7% specificity. The IL-1R2 upmodulation measured in HC, IP, WMC, and T patients is reported in Figure 4D, right plot, which also shows the cutoff value. MO IL-1R2 upmodulation was significantly higher in IP and WMC patients than HC (*p* < 0.0001 and *p* < 0.002, one-way ANOVA and Tukey’s test, respectively). Two out of five T patients had an IL-1R2 expression level above the cutoff.

## 3. Discussion

Although several studies have reported the usefulness of FC assays for the rapid identification of bacterial and viral infections [17,18,19,20,21,22,23,24,25,26,32,33,35,36], their use in routine clinical microbiology practice is still limited. We have argued that the acceptance and incorporation of FC assays into standard paradigms of care and the management of febrile patients could be facilitated by minimizing subjectivity and technicalities. Thus, we developed an FC assay that utilizes a straightforward gating procedure and data analysis to aid novice users in detecting the presence of a pathogen. Furthermore, we incorporated two phenotypic markers of MO as indicators of the patient’s immune system status in the assay. We deliberately included febrile patients across multiple clinical syndromes and pathogen species to underline the wide applicability of the present FC assay.

The present FC assay identifies bacterial infection by the increased expression level of CD64 PMN, in line with previous reports [20,21,22,23,24,25,32,35]. To this end, based on existing FC assays [20,21,22,23,24,25,32,35], we developed a PMN index that has shown excellent performance in terms of specificity and sensitivity due to the high efficiency of the monoclonal antibody used for the detection of CD64. Importantly, this good performance was observed in a cohort of unselected consecutive febrile patients with a wide range of disease severity, highlighting the ability of this FC assay to quickly identify bacterial infections in the context of general clinical practice, suggesting that the high PMN index in febrile patients should encourage treating physicians to request an immediate microbiological investigation to arrive at a definitive diagnosis.

In line with previously published FC assays [17,18,19,20,21,22,24,25,26], the present FC assay detects viral infection as MO CD169 upmodulation. Although the assay performance was good, it was not as satisfactory as that for detecting bacterial infection. We hypothesize that the fortuitous inclusion of subjects with asymptomatic viral infection, a fairly common occurrence in the generally “healthy” population, may have adversely affected the performance of the assay. Performance was somewhat lower than reported in some previous studies [33,42,43]. We can only conjecture as to why our results differ. However, certain comparative features are worth noting. The patient population in these studies was predominantly patients with SARS-CoV-2 who typically have higher levels of MO CD169 expression than those infected by other viral species [33]. In addition, the level of MO CD169 expression is associated with disease severity [42,43]. The present study included more patients infected with various viral species than SARS-CoV-2 and only two critically ill SARS-CoV-2 patients.

The present FC assay incorporated the MO HLA-DR expression level assessment as a surrogate indicator of immune fitness. We observed that MO HLA-DR downmodulation characterized most patients with microbiologically confirmed bacterial and viral infection, in line with the current literature [22,44,45,46,47]. Notably, we found MO HLA-DR downmodulation above the cutoff in a sizeable proportion of the patients in which microbiological confirmation was not sought and observed that half of these patients had a PMN index and/or MO CD169 above the cutoff, arguing for the presence of an overlooked pathogen. Notably, the MO HLA-DR expression level measured by the present FC assay was well matched to that obtained with calibrated beads, which is consensually accepted as a reliable method for estimating the numbers of HLA-DR molecules on the MO membrane [37]. This highlights the reliability of the present FC assay in the MO HLA-DR modulation measure.

To our knowledge, the presence of MO IL-1R2 in patients with infectious diseases has been described in only one study on patients with severe bacterial infections [31]. Here, we found MO IL-1R2 in the vast majority of patients with microbiologically confirmed bacterial and viral infection, an unexpected result as most of the patients were not seriously ill. IL-1R2 participates in the regulation of the inflammatory response in competition with IL-1R1 to bind IL-1 and restore homeostasis [29,30]. However, IL-1 is also an established cause of fever due to the stimulation of the hypothalamus [48]. Since all the patients included in the study had a fever, we can infer the generalized increase in MO IL-1R2 expression level to be a common phenomenon that intervenes to counteract the biological activity of IL-1, even in mild infections. Studies focusing on the relationship between MO IL-1R2 expression level and plasma IL-1 level are needed to clarify this point. The same study [31] showed that the upmodulation of MO IL-1R2 concomitantly with the downmodulation of MO HLA-DR indicated a dysregulated immune response to bacterial infection and a negative prognosis. We did not find any significant correlation between the two markers in the entire cohort of infected patients, probably due to the low number of critical patients included in our study. However, we observed MO IL-1R2 upmodulation concomitant to MO HLA-DR downmodulation in two infected febrile patients who also had advanced cancers, thus supporting the efficiency of this FC assay in stratifying the risk of infected patients. Further studies may be warranted to confirm the utility of MO HLA-DR and MO IL-1R2 modulation in larger populations of patients with infectious diseases in relation to the presence or absence of an underlying (severe) disease.

This study included a small group of patients with uveal melanoma with no clinical and laboratory evidence of infection who were treated with the therapeutic antibody tebentafusp, a bispecific antibody that docks T cells to glycoprotein 100 (gp100) on melanoma cells and induces the release of several inflammatory cytokines [49] to explore the activity of cytokines on the PMN and MO surface markers we explored here. We found that the surface markers were randomly modulated in some patients, thus representing a warning in the use of the present FC assay for detecting bacterial and viral infection in patients receiving drugs that potentially induce cytokine production. In addition, an early study showed that the level of PMN CD64 expression tended to be elevated in cancer patients without active infection [50].

The present FC assay has some limitations. Firstly, similar to other FC assays, it cannot definitively identify the specific bacterial and viral species involved in an infection. Secondly, the assessment of monocyte MO HLA-DR expression levels can be hindered in conditions such as profound B-cell lymphopenia or when there is a substantial presence of circulating neoplastic B cells. Moreover, each laboratory is required to create its own dataset of healthy control samples due to the absence of established reference intervals, which can be a significant barrier to standardization. Looking ahead, it is expected that the development of standardized and commercially available antibody combinations, ideally in a dried format that allows for easier handling and preparation, will enhance the adoption of this assay in clinical microbiology laboratories. Such advancements could also support efforts for harmonization across different healthcare centers.

Lastly, there remains an urgent need for further research, particularly in larger population studies, to evaluate the assay’s capability to differentiate between infectious and non-infectious fever. This is especially relevant for patients with autoimmune diseases, where alterations in polymorphonuclear (PMN) and monocyte surface markers have been observed without clear signs of infection [50,51,52,53]. Addressing these challenges will be crucial for improving the diagnostic accuracy of the present FC assay in clinical practice.

## 4. Materials and Methods

### 4.1. Patient Accrual and Ethics Statement

We prospectively recruited 74 patients (48 males: median age 67.5 years, 25th and 75th percentile 47.25–74.75, respectively; 26 females: median age 65 years, 25th and 75th percentile 27.25–79.5, respectively) from the emergency department, internal medicine department, and surgical department of Policlinico Universitario A. Gemelli, IRCCS, Rome, Italy. The study included 8 non-infected patients affected by uveal melanoma that underwent immunotherapy with tebentafusp, a bispecific antibody engaging T cells with glycoprotein 100 (gp100) on melanoma cells that induces cytokine release syndrome (CRS) [49], and 24 subjects without either clinical or laboratory signs of infection, referred to as HC and matched for age and sex.

This was a not-for-profit, observational, single-center study approved by the internal Institutional Ethics Committee (approval code: protocol 0039313/21, SepsiFlow, Prot ID 4537; date of approval, 9 November 2021). This study was conducted in accordance with the Declaration of Helsinki. Written informed consent was obtained from each participant or legal guardian, as applicable.

### 4.2. Study Population

Inclusion criteria were peak fever > 37.5 °C since onset of symptoms and clinical suspicion of an acute infectious disease based on medical history, physical examination, complete blood count, and chemistry panel on the judgement of the treating physician. Patients were consecutively recruited, irrespective of underlying disease and reason for hospital admission. Empiric antibiotic treatment was not an exclusion criterion. Blood was collected within 4 days since onset of symptoms. Patients were retrospectively stratified according to whether bacterial and viral infection was confirmed by appropriate microbiological tests. Cultures from blood, urine, bronchoalveolar lavage, and stool were performed using aerobic and anaerobic bottle (BACT/ALERT^®^ VIRTUO bioMérieux, Marcy l’Etoile, France) before starting empiric antibiotics, unless delayed initiation of antibiotics could worsen mortality. Bottles were incubated up to five days or until they signaled positive. In this case, broth aliquots were collected for standard identification studies, which entailed Gram staining, direct analysis in the Bruker MALDI BioTyper (Bruker Daltonik GmbH, Leipzig, Germany), and solid-medium subcultures. After isolation from the cultures, bacteria were identified by MALDI BioTyper analysis at log (score) values > 2.0. [54]. The respiratory specimens were tested with the FilmArray^®^ Pneumonia Plus Panel (FAPP) (Biofire, BioMérieux Marcy l’Etoile, France), which detects a panel including 15 typical bacteria, 3 atypical bacteria, 7 antimicrobial resistance (AMR) genes, and 9 viruses. For SARS-CoV-2 and other respiratory virus detection, including Rhinoviruses (RVs), respiratory Enteroviruses (EVs), and respiratory syncytial virus (RSV), the Allplex SARS-CoV-2 and AllplexTM Respiratory Pannel Assays (Seegene, Seoul, Republic of Korea) were utilized. For the detection of Cytomegalovirus (CMV) and Epstein–Barr Virus (EBV), the Artus CMV and EBV QS-RGQ Kits (QIAGEN, Venlo, The Netherlands), respectively, were utilized. For the detection of Herpesvirus type 1 (HSV-1) and type 2 (HSV-2), the RealStar^®^ alpha Herpesvirus kit (Altona Diagnostics, Hamburg, Germany) was utilized.

### 4.3. Sample Collection

Peripheral blood samples were collected by venipuncture into an EDTA-containing vacutainer blood collection tube. Preliminary experiments showed that marker expression underwent time-associated changes that varied among different markers when the sample processing did not start within 2 h from blood draw. Thus, all flow data were generated within this time frame.

### 4.4. Monoclonal Antibodies and Staining Procedure

Even careful handling of PMN ex vivo is fraught with unwanted heterogeneity and alterations that can potentially reduce assay reproducibility and alter biological conclusions. Thus, as the present FC assay was designed to be employed in routine settings, we felt it important to exclude sample logistics that affected PMN viability and the FC assay results. To this end, we performed a series of real-life FC experiments. The data showed that variable proportions of PMN underwent apoptosis during blood collection, in line with a previous report [38], although membrane damage did not affect FC assay data (Supplementary Information).

We adapted the commercially available 3-color antibody combination IOTest Myeloid Activation kit CD169-PE/HLA-DR-APC/CD64-PB Antibody Cocktail™ (Beckman Coulter, Brea, CA, USA) [55]. We reasoned that a pre-formulated 3-color antibody combination would have reduced the error-prone pipetting of liquid reagent and ensured superior workflow efficiency and more reliable FC results than dispensing each reagent individually. However, in the preamble to the study, we tested 18 HC and 33 microbiologically confirmed bacterial infection patients and found that the CD64-PB signal was fairly dim, conceivably reducing its ability to identify bacterial infection. Thus, we substituted the brighter isoclonic CD64-PC7 conjugate (Beckman Coulter) for the weak CD64-PB conjugate. To this end, we dispensed 2 μL of the CD64-PC7 Moab to the whole blood sample 20 min before carrying on with staining with the IOTest Myeloid Activation kit CD169-PE/HLA-DR-APC/CD64-PB Antibody Cocktail™. The amount of CD64-PC7 Moab necessary to saturate CD64 binding sites was determined empirically and was consistent throughout the study. IL-1R2-FITC Moab (Invitrogen, Waltham, MA, USA) and CD19-PC7 Moab (Beckman Coulter) were pipetted along with the IOTest Myeloid Activation kit CD169-PE/HLA-DR-APC/CD64-PB Antibody Cocktail™. This Moab panel had minimum spectral overlap and no cross-laser excitation so as to simplify technicalities. In fact, the only spectral overlap with a potential detrimental effect on the data analysis was due to the CD64-PC7 signal entering the PE-CD169 channel, which could be very easily detected and compensated via visual inspection, even by inexperienced users. Moabs were added to 50 μL whole blood in polypropylene non-sterile flow cytometry 5 mL round-bottom tube and mixed by gently pipetting. Samples were incubated at room temperature in the dark for 20 min. VersaLyse™ (Beckman Coulter) was used for the lysis of red blood cells following the manufacturer’s instructions. Samples were immediately run on the cytometer. FC analysis was performed on a DxFLEX flow cytometer (Beckman Coulter) equipped with 488 nm, 638 nm, and 405 nm lasers, except for the viability experiments during the set-up procedure that were performed on a CytoFLEX LX also equipped with 355 nm laser for DAPI excitation (Supplementary Information). Scatter and fluorescence signals were determined for each cell and stored in list-mode data files in FCS 3.0 format. Samples were acquired at ~500 events/s. An acquisition region that included MO was established on SSC/CD64 dot plot to collect at least 6 × 10^3^ cells in each run. The cytometer was calibrated daily using Daily QC Fluorospheres (Beckman Coulter). Some experiments were carried out to compare the HLA-DR expression measured by the present FC assay with that being measured by the BD Quantibrite™ Anti–HLA-DR/Anti-Monocyte kit (BD Biosciences, Franklin Lakes, NJ, USA). To this end, we selected six representative samples with various HLA-DR expression levels. Sample preparation and flow cytometric analysis were carried out as detailed in the technical data sheet included in the kit. Flow data were first generated and analyzed by an inexperienced user and then independently checked for consistency by an expert investigator using the CytExpert™ Software 2.0 (Beckman Coulter). Users were both blinded to the clinical data of patients to minimize biases in data analysis.

### 4.5. Statistics

Study samples were assigned a designation of infection—either bacterial or viral—or no infection. Box whiskers and descriptive statistics were generated for each biomarker. Welch’s ANOVA for more than two groups was used to test for differences between group means followed by Tukey’s multiple comparison test. Receiver operating characteristic (ROC) curves were constructed and both Area Under the Curve (AUC) and cutoffs for the best balance of sensitivity and specificity were calculated by the likelihood ratio test. Positive predictive value (PPV) and negative predictive value (NPV) were also calculated.

## Data Availability

The original contributions presented in this study are included in the article/Supplementary Materials. Further inquiries can be directed to the corresponding author.

## Supplementary Materials

The following supporting information can be downloaded at: https://www.mdpi.com/article/10.3390/ijms252111632/s1.

## References

[1] Baron E.J., Miller J.M., Weinstein M.P., Richter S.S., Gilligan P.H., Thomson R.B., Bourbeau P., Carroll K.C., Kehl S.C., Dunne W.M. (2013). A Guide to Utilization of the Microbiology Laboratory for Diagnosis of Infectious Diseases: 2013 Recommendations by the Infectious Diseases Society of America (IDSA) and the American Society for Microbiology (ASM). Clin. Infect. Dis..

[2] Faix J.D. (2013). Biomarkers of sepsis. Crit. Rev. Clin. Lab. Sci..

[3] Woodhead M., Blasi F., Ewig S., Garau J., Huchon G., Ieven M., Ortqvist A., Schaberg T., Torres A., van der Heijden G. (2011). Guidelines for the management of adult lower respiratory tract infections—Full version. Clin. Microbiol. Infect..

[4] Laxminarayan R., Duse A., Wattal C., Zaidi A.K.M., Wertheim H.F.L., Sumpradit N., Vlieghe E., Hara G.L., Gould I.M., Goossens H. (2013). Antibiotic resistance—The need for global solutions. Lancet Infect. Dis..

[5] Machen A., Drake T., Wang Y.F. (2014). Same Day Identification and Full Panel Antimicrobial Susceptibility Testing of Bacteria from Positive Blood Culture Bottles Made Possible by a Combined Lysis-Filtration Method with MALDI-TOF VITEK Mass Spectrometry and the VITEK2 System. PLoS ONE.

[6] Connell T.G., Rele M., Cowley D., Buttery J.P., Curtis N. (2007). How Reliable Is a Negative Blood Culture Result? Volume of Blood Submitted for Culture in Routine Practice in a Children’s Hospital. Pediatrics.

[7] Rhedin S., Lindstrand A., Rotzén-Östlund M., Tolfvenstam T., Öhrmalm L., Rinder M.R., Zweygberg-Wirgart B., Ortqvist A., Henriques-Normark B., Broliden K. (2014). Clinical Utility of PCR for Common Viruses in Acute Respiratory Illness. Pediatrics.

[8] Murphy K., Weaver C. (2016). Janeway’s Immunobiology.

[9] Nimmerjahn F., Ravetch J.V. (2006). Fcγ Receptors: Old Friends and New Family Members. Immunity.

[10] Hoffmeyer F., Witte K., Schmidt R.E. (1997). The high-affinity FcγRI on PMN: Regulation of expression and signal transduction. Immunology.

[11] Wagner C., Deppisch R., Denefleh B., Hug F., Andrassy K., Hänsch G.M. (2003). Expression Patterns of the Lipopolysaccharide Receptor CD14, and the Fcγ Receptors CD16 and CD64 on Polymorphonuclear Neutrophils: Data from Patients with Severe Bacterial Infections and Lipopolysaccharide-Exposed Cells. Shock.

[12] Repp R., Valerius T., Sendler A., Gramatzki M., Iro H., Kalden J., Platzer E. (1991). Neutrophils express the high affinity receptor for IgG (Fc gamma RI, CD64) after in vivo application of recombinant human granulocyte colony-stimulating factor. Blood.

[13] Kerst J., de Haas M., van der Schoot C., Slaper-Cortenbach I., Kleijer M., Borne A.v.D., van Oers R. (1993). Recombinant granulocyte colony-stimulating factor administration to healthy volunteers: Induction of immunophenotypically and functionally altered neutrophils via an effect on myeloid progenitor cells. Blood.

[14] Uchil P.D., Pi R., Haugh K.A., Ladinsky M.S., Ventura J.D., Barrett B.S., Santiago M.L., Bjorkman P.J., Kassiotis G., Sewald X. (2019). A Protective Role for the Lectin CD169/Siglec-1 against a Pathogenic Murine Retrovirus. Cell Host Microbe.

[15] Shinde P.V., Xu H.C., Maney S.K., Kloetgen A., Namineni S., Zhuang Y., Honke N., Shaabani N., Bellora N., Doerrenberg M. (2018). Tumor Necrosis Factor-Mediated Survival of CD169^+^ Cells Promotes Immune Activation during Vesicular Stomatitis Virus Infection. J. Virol..

[16] Kim W.-K., McGary C.M., Holder G.E., Filipowicz A.R., Kim M.M., Beydoun H.A., Cai Y., Liu X., Sugimoto C., Kuroda M.J. (2015). Increased Expression of CD169 on Blood Monocytes and Its Regulation by Virus and CD8 T Cells in Macaque Models of HIV Infection and AIDS. AIDS Res. Hum. Retroviruses.

[17] Van der Kuyl A.C., Burg R.v.D., Zorgdrager F., Groot F., Berkhout B., Cornelissen M. (2007). Sialoadhesin (CD169) Expression in CD14+ Cells Is Upregulated Early after HIV-1 Infection and Increases during Disease Progression. PLoS ONE.

[18] Michlmayr D., Kim E.-Y., Rahman A.H., Raghunathan R., Kim-Schulze S., Che Y., Kalayci S., Gümüş Z.H., Kuan G., Balmaseda A. (2020). Comprehensive Immunoprofiling of Pediatric Zika Reveals Key Role for Monocytes in the Acute Phase and No Effect of Prior Dengue Virus Infection. Cell Rep..

[19] Bedin A.-S., Makinson A., Picot M.-C., Mennechet F., Malergue F., Pisoni A., Nyiramigisha E., Montagnier L., Bollore K., Debiesse S. (2021). Monocyte CD169 Expression as a Biomarker in the Early Diagnosis of Coronavirus Disease 2019. J. Infect. Dis..

[20] Minutolo A., Petrone V., Fanelli M., Iannetta M., Giudice M., Belkacem I.A., Zordan M., Vitale P., Rasi G., Sinibaldi-Vallebona P. (2021). High CD169 Monocyte/Lymphocyte Ratio Reflects Immunophenotype Disruption and Oxygen Need in COVID-19 Patients. Pathogens.

[21] Comins-Boo A., Gutiérrez-Larrañaga M., Roa-Bautista A., Foz S.G., García M.R., López E.G., Ventura J.I., Fariñas-Álvarez M.C., Segundo D.S., Hoyos M.L. (2021). Validation of a Quick Flow Cytometry-Based Assay for Acute Infection Based on CD64 and CD169 Expression. New Tools for Early Diagnosis in COVID-19 Pandemic. Front. Med..

[22] Gatti A., Fassini P., Mazzone A., Rusconi S., Brando B., Mistraletti G. (2023). Kinetics of CD169, HLA-DR, and CD64 expression as predictive biomarkers of SARS-CoV2 outcome. J. Anesth. Analg. Crit. Care.

[23] Park J., Dean L.S., Jiyarom B., Gangcuangco L.M., Shah P., Awamura T., Ching L.L., Nerurkar V.R., Chow D.C., Igno F. (2023). Elevated circulating monocytes and monocyte activation in COVID-19 convalescent individuals. Front. Immunol..

[24] Affandi A.J., Olesek K., Grabowska J., Twilhaar M.K.N., Rodríguez E., Saris A., Zwart E.S., Nossent E.J., Kalay H., de Kok M. (2021). CD169 Defines Activated CD14^+^ Monocytes with Enhanced CD8^+^ T Cell Activation Capacity. Front. Immunol..

[25] Bourgoin P., Biéchelé G., Belkacem I.A., Morange P., Malergue F. (2020). Role of the interferons in CD64 and CD169 expressions in whole blood: Relevance in the balance between viral- or bacterial-oriented immune responses. Immun. Inflamm. Dis..

[26] Sakumura N., Yokoyama T., Usami M., Hosono Y., Inoue N., Matsuda Y., Tasaki Y., Wada T. (2023). CD169 expression on monocytes as a marker for assessing type I interferon status in pediatric inflammatory diseases. Clin. Immunol..

[27] Volk H.D., Reinke P., Krausch D., Zuckermann H., Asadullah K., Müller J.M., Döcke W.D., Kox W.J. (1996). Monocyte deactivation-rationale for a new therapeutic strategy in sepsis. Intensiv. Care Med..

[28] Monneret G., Lepape A., Voirin N., Bohé J., Venet F., Debard A.-L., Thizy H., Bienvenu J., Gueyffier F., Vanhems P. (2006). Persisting low monocyte human leukocyte antigen-DR expression predicts mortality in septic shock. Intensiv. Care Med..

[29] Garlanda C., Riva F., Bonavita E., Mantovani A. (2013). Negative regulatory receptors of the IL-1 family. Semin. Immunol..

[30] Zhang Y., Liu K., Guo M., Yang Y., Zhang H. (2024). Negative regulator IL-1 receptor 2 (IL-1R2) and its roles in immune regulation of autoimmune diseases. Int. Immunopharmacol..

[31] Reyes M., Filbin M.R., Bhattacharyya R.P., Billman K., Eisenhaure T., Hung D.T., Levy B.D., Baron R.M., Blainey P.C., Goldberg M.B. (2020). An immune-cell signature of bacterial sepsis. Nat. Med..

[32] Bourgoin P., Soliveres T., Ahriz D., Arnoux I., Meisel C., Unterwalder N., Morange P.-E., Michelet P., Malergue F., Markarian T. (2019). Clinical research assessment by flow cytometry of biomarkers for infectious stratification in an Emergency Department. Biomark. Med..

[33] Bourgoin P., Soliveres T., Barbaresi A., Loundou A., Belkacem I.A., Arnoux I., Bernot D., Loosveld M., Morange P., Michelet P. (2021). CD169 and CD64 could help differentiate bacterial from COVID-19 or other viral infections in the Emergency Department. Cytom. Part A.

[34] Perry S.E., Mostafa S.M., Wenstone R., Shenkin A., McLaughlin P.J. (2003). Is low monocyte HLA-DR expression helpful to predict outcome in severe sepsis?. Intensiv. Care Med..

[35] Hiesmayr M.J., Spittler A., Lassnigg A., Berger R., Laufer G., Kocher A., Artemiou O., Boltz-Nitulescu G., Roth E. (1999). Alterations in the number of circulating leucocytes, phenotype of monocyte and cytokine production in patients undergoing cardiothoracic surgery. Clin. Exp. Immunol..

[36] Oczenski W., Krenn H., Jilch R., Watzka H., Waldenberger F., Schwarz S., Fitzgerald R.D., Köller U. (2003). HLA-DR as a marker for increased risk for systemic inflammation and septic complications after cardiac surgery. Intensiv. Care Med..

[37] Döcke W.-D., Höflich C., A Davis K., Röttgers K., Meisel C., Kiefer P., Weber S.U., Hedwig-Geissing M., Kreuzfelder E., Tschentscher P. (2005). Monitoring Temporary Immunodepression by Flow Cytometric Measurement of Monocytic HLA-DR Expression: A Multicenter Standardized Study. Clin. Chem..

[38] Krabbe J., Beilmann V., Alamzad-Krabbe H., Böll S., Seifert A., Ruske N., Kraus T., Martin C. (2020). Blood collection technique, anticoagulants and storing temperature have minor effects on the isolation of polymorphonuclear neutrophils. Sci. Rep..

[39] Mosiman V.L., Patterson B.K., Canterero L., Goolsby C.L. (1997). Reducing Cellular Autofluorescence in Flow Cytometry: An In Situ Method. Cytom. J. Int. Soc. Anal. Cytol..

[40] Kwok A.J., Allcock A., Ferreira R.C., Cano-Gamez E., Smee M., Burnham K.L., Zurke Y.-X., Novak A., Darwent M., Emergency Medicine Research Oxford (EMROx) (2023). Neutrophils and emergency granulopoiesis drive immune suppression and an extreme response endotype during sepsis. Nat. Immunol..

[41] McMahan C., Slack J., Mosley B., Cosman D., Lupton S., Brunton L., Grubin C., Wignall J., Jenkins N., Brannan C. (1991). A novel IL-1 receptor, cloned from B cells by mammalian expression, is expressed in many cell types. EMBO J..

[42] Doehn J.-M., Tabeling C., Biesen R., Saccomanno J., Madlung E., Pappe E., Gabriel F., Kurth F., Meisel C., Corman V.M. (2021). CD169/SIGLEC1 is expressed on circulating monocytes in COVID-19 and expression levels are associated with disease severity. Infection.

[43] Vetter P., Eberhardt C.S., Meyer B., Murillo P.A.M., Torriani G., Pigny F., Lemeille S., Cordey S., Laubscher F., Vu D.-L. (2020). Daily Viral Kinetics and Innate and Adaptive Immune Response Assessment in COVID-19: A Case Series. mSphere.

[44] Hotchkiss R.S., Monneret G., Payen D. (2013). Immunosuppression in sepsis: A novel understanding of the disorder and a new therapeutic approach. Lancet Infect. Dis..

[45] Lukaszewicz A.-C., Grienay M., Resche-Rigon M., Pirracchio R., Faivre V., Boval B., Payen D. (2009). Monocytic HLA-DR expression in intensive care patients: Interest for prognosis and secondary infection prediction. Crit. Care Med..

[46] Chen Y., Zhuang Y., Peng H., Chen Y., Zhou S. (2017). Dynamic monitoring of monocyte HLA-DR expression for the diagnosis prognosis and prediction of sepsis. Front. Biosci..

[47] Monneret G., Venet F. (2016). Sepsis-induced immune alterations monitoring by flow cytometry as a promising tool for individualized therapy. Cytom. Part B Clin. Cytom..

[48] Dinarello C.A. (2018). Overview of the IL-1 family in innate inflammation and acquired immunity. Immunol. Rev..

[49] Boudousquie C., Bossi G., Hurst J.M., Rygiel K.A., Jakobsen B.K., Hassan N.J. (2017). Polyfunctional response by ImmTAC (IMCgp100) redirected CD8^+^ and CD4^+^ T cells. Immunology.

[50] Comolli G., Torchio M., Lenta E., Franceschetti B., Chiesa A., Calarota S.A., Baldanti F., Scudeller L., Marone P., Danova M. (2015). Neutrophil CD64 expression: A reliable diagnostic marker of infection in advanced cancer patients?. New Microbiol..

[51] Jalava-Karvinen P., Hohenthal U., Laitinen I., Kotilainen P., Rajamäki A., Nikoskelainen J., Lilius E.-M., Nuutila J. (2009). Simultaneous quantitative analysis of FcγRI (CD64) and CR1 (CD35) on neutrophils in distinguishing between bacterial infections, viral infections, and inflammatory diseases. Clin. Immunol..

[52] Steinbach F., Henke F., Krause B., Thiele B., Burmester G.-R., Hiepe F. (2000). Monocytes from systemic lupus erythematous patients are severely altered in phenotype and lineage flexibility. Ann. Rheum. Dis..

[53] York M.R., Nagai T., Mangini A.J., Lemaire R., van Seventer J.M., Lafyatis R. (2007). A macrophage marker, siglec-1, is increased on circulating monocytes in patients with systemic sclerosis and induced by type i interferons and toll-like receptor agonists. Arthritis Rheum..

[54] Fiori B., D’Inzeo T., Giaquinto A., Menchinelli G., Liotti F.M., de Maio F., De Angelis G., Quaranta G., Nagel D., Tumbarello M. (2016). Optimized Use of the MALDI BioTyper System and the FilmArray BCID Panel for Direct Identification of Microbial Pathogens from Positive Blood Cultures. J. Clin. Microbiol..

[55] Bourgoin P., Lediagon G., Arnoux I., Bernot D., Morange P.-E., Michelet P., Malergue F., Markarian T. (2020). Flow Cytometry Evaluation of Infection-Related Biomarkers in Febrile Subjects in the Emergency Department. Futur. Microbiol..

