# Supplementary material for: A New Easy-to-Perform Flow Cytometry Assay for Determining Bacterial- and Viral-Infection-Induced Polymorphonuclear Neutrophil and Monocyte Membrane Marker Modulation in Febrile Patients
Source: Int J Mol Sci. 2024 Oct 29;25(21):11632. doi: 10.3390/ijms252111632 (PMC11547050; doi:10.3390/ijms252111632)
Supplement: Supplementary file 1 [file ijms-25-11632-s001.zip › ijms-3267455-supplementary.pdf]

Sample processing-induced PMN apoptosis does not affect FC assay results.

The experiments reported here used a CytoFLEX LX flow cytometer (Beckman Coulter) equipped with 488 nm, 638 nm, 405 nm and 355 nm lasers. During the study, two PMN populations were often differentiated according to the relative intensity of the two scatter signals, i.e., SSC^{high}/FSC^{low} and SSC^{low}/FSC^{high}) (Fig 1A). These peculiar scatter signals in cells of leukocyte origin usually indicate cell damage and are reminiscent of an early report describing the presence of apoptotic PMN defined by similar scatter signals in lysed human whole blood samples in various disease settings and in healthy donors [38]. Therefore, we suspected that SSC^{high}/FSC^{low} PMN represented nonviable cells and thus a potential source of artefacts because of increased autofluorescence and non-specific antibodies binding. We found that the SSC^{high}/FSC^{low} PMN population did indeed represent PMN with altered cell membrane integrity, as they admitted the cell-impermeant dye DAPI (Fig 1B). This PMN population was observed irrespective of the modality of blood sampling, i.e., Vacutainer® or aspirating syringe and needle gauge, leading to the conclusion that the SSC^{high}/FSC^{low} PMN represented PMN that had been inadvertently damaged in some way during sample handling. In FC assays, data analysis should be limited to intact cells. In principle, the SSC^{high}/FSC^{low} PMN population could be excluded by drawing a polygonal region around the SSC^{low}/FSC^{high} PMN intact population. However, since the present FC assay was designed primarily for inexperienced users, we decided to avoid adding this initial gating in the assay. To this end, we determined whether the interpretation of the data would be affected by the inclusion of damaged PMN in the analysis. The CD64 expression level was identical in the intact and damaged PMN in healthy controls (very low CD64 expression level, Fig. 1C) and bacterial infection patients (high CD64 expression level, Fig1 D) demonstrating that both PMN populations participate equally in CD64 modulation. Figure 1E shows that damaged and intact PMN do not stain differently for CD169, an important aspect since the present FC assay uses PMN autofluorescence to set the boundary for CD169 positive MO.

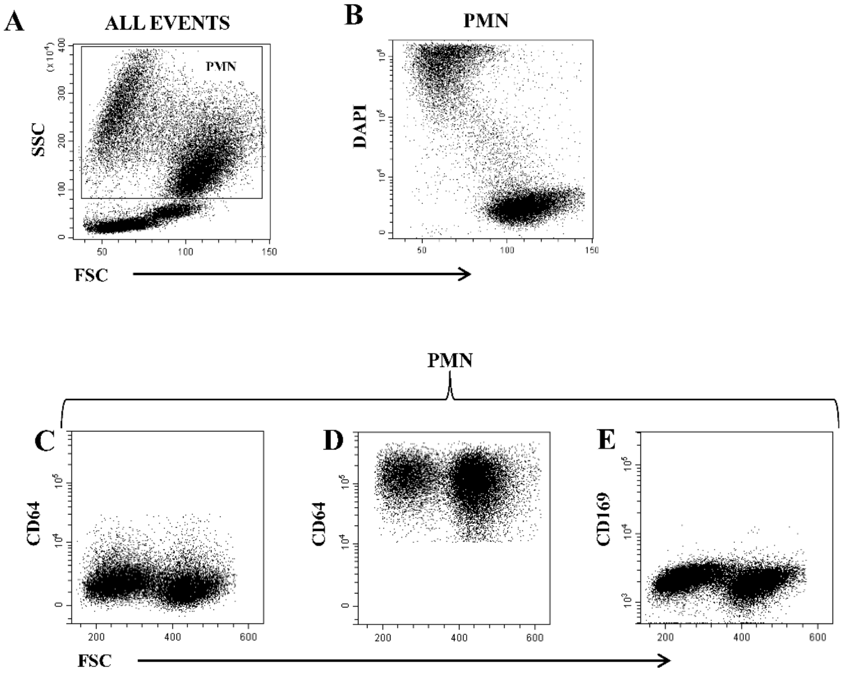

Figure S1. Damaged PMN in the sample does not affect flow data. A) SSC/FSC dual parameter plot showing two distinct PMN populations defined as SSC^{high}/FSC^{low} and SSC^{low}/FSC^{high} . B) FSC/DAPI dual parameter plot showing that the SSC^{high}/FSC^{low} PMN population admits the vital dye. C) Intact and damaged PMN show identical autofluorescence in CD64 channel in healthy subjects. D) Intact and damaged PMN show indistinguishable CD64 upmodulation in patients with microbial infection. E) Intact and damaged PMN show identical autofluorescence in CD169 channel. Plots C-E derive from the PMN region shown in A).

All plots derive from a first level gating on forward scatter (FSC) versus side scatter (SSC) to exclude most erythrocyte ghosts and debris (not shown).
